# Analysis of Spatial, Binaural, and Better-Ear Benefits for Different Degrees of Hearing Loss Using a Binaural Speech Intelligibility Model

**DOI:** 10.1177/23312165261418655

**Published:** 2026-02-12

**Authors:** Saskia Röttges, Christopher Hauth, Kirsten C. Wagener, Thomas Brand

**Affiliations:** 1 Medizinische Physik, 597451 Carl von Ossietzky Universität, Oldenburg, Germany; 2 Cluster of Excellence 597459 “Hearing4all”, Oldenburg, Germany; 3 Hörzentrum Oldenburg gGmbH, Oldenburg, Germany

**Keywords:** binaural, speech recognition, model, hearing impaired, speech perception

## Abstract

This study analyzed speech recognition thresholds (SRTs) in noise of 738 listeners with different degrees of hearing loss, including normal hearing. Speech was presented from the front, while noise was presented from either the front or from +90° or −90° azimuth, corresponding to the ear with the worse hearing threshold. The latter condition was tested binaurally and monaurally (using the only better ear (BE)). Listeners showed larger variance in SRTs than in spatial, binaural, and BE benefits, regardless of their hearing loss. A model consisting of a blind equalization-cancellation (EC) front-end and the non-blind speech intelligibility index (SII, ANSI S3.5-1997) was used to predict the measured SRTs. This model used a pure-tone audiogram to simulate hearing loss. We evaluated whether an individual suprathreshold component improves the model's prediction accuracy. This component was implemented in two ways: as an individual reference SII value (from the SRT of the S_0_N_0_ situation) and as an individually increased external noise. Both methods improved prediction accuracy for the S_0_N_90_ conditions but had no effect on spatial or binaural release from masking, nor on BE listening. No evidence was found that other available parameters would improve the prediction accuracy.

## Introduction

The speech recognition threshold (SRT) in noise—the signal-to-noise ratio (SNR) at which 50% of the words in a sentence are recognized—is a characteristic measure of human speech recognition performance and can be used to assess speech intelligibility for normal-hearing (NH) and hearing-impaired (HI) listeners. Various studies have described higher SRTs for HI listeners compared to NH listeners (Beutelmann & Brand, 2006; Humes, 1996; Smits & Festen, 2011; Wagener & Brand, 2005), and the extent of this difference is typically related to the listener's hearing loss. In speech-in-noise conditions, the human auditory system is able to exploit the interaural level difference (ILD) and the interaural time difference (ITD) of the input signals. Better-ear listening (BEL) occurs when target speech and noise differ in their ILDs and thus one ear exhibits a better SNR. Binaural release from masking (BRM) occurs when target speech and noise differ in their ITDs (or IPDs), which enables binaural processing for improving the effective SNR (see model descriptions below). Spatial release from masking (SRM) occurs as a consequence of the combined BEL and BRM due to differences in ILDs and ITDs caused by the different spatial positions of target speech and noise. In general, the release from masking can be affected by hearing loss (Bronkhorst & Plomp, 1989).

There are different approaches to modeling binaural speech intelligibility. One approach (Geravanchizadeh & Fallah, 2015) combines an auditory periphery model with an equalization-cancellation (EC) mechanism (Durlach, 1963). The EC mechanism is characterized as a blind model, as it operates on mixed speech and signals, and the model's back-end features a dynamic time-warp speech recognizer (Sakoe & Chiba, 1978), which aligns the input signal with the model's reference templates, allowing time-scale adjustments to find the best match. This approach is primarily applicable to scenarios with negative SNRs, as conventional EC processing was designed to eliminate the dominant signal. Another approach (Tang et al., 2018) combines a non-blind speech intelligibility back-end with a source separation algorithm. That approach assumes that there is only one masking source and that the target source is situated in front of two microphones. A source separation algorithm estimates the speech signal and the noise signal, which can then be further analyzed with multiple speech intelligibility back-ends.

Another approach to modeling binaural intelligibility (Lavandier & Culling, 2010) is a combination of better-ear listening and binaural release from masking. Binaural masking level differences were calculated to simulate the binaural effects. The monaural effects were predicted by using the excitation pattern of the noise. Weights according to the speech intelligibility index (SII, ANSI S3.5-1997) were subsequently applied to both aspects. SRTs were measured in headphone experiments. The interferer was placed in a virtual room with different room sizes, wall absorption, azimuth angle and distance to the listener. However, the target sound was always anechoic. The model achieved a correlation between measured and modeled SRTs of 0.95–0.97. This model was further developed to incorporate more realistic conditions (Lavandier et al., 2012), such as multiple stationary noises (achieving a correlation between measured and modeled SRTs of 0.95–0.99) or non-stationary noises (Collin & Lavandier, 2013). A further development of this model for speech intelligibility predictions of hearing-impaired listeners (Vicente et al., 2020, 2021) is explained in more detail below.

In this study, we use the Binaural Speech Intelligibility Model (BSIM), introduced by Beutelmann and Brand (2006) and updated by Hauth et al. (2020), to determine how well SRTs can be predicted in binaural listening conditions for a large set of listeners with normal or impaired hearing. Doing this, we also want to test whether the model is able to identify outliers, that is, listeners whose SRTs deviate significantly from the predictions, and whether these outliers show any striking features in their hearing parameters.

In order to make predictions for hearing-impaired listeners, the scope of the hearing loss has to be considered. One concept for modeling the reduced audibility caused by hearing loss is to exclude all signal components falling below the hearing threshold in a given frequency band from the model's representation of the signal. This method is used, for example, by Cooke (2006) and Hülsmeier et al. (2020). An alternative way to model audibility is to add a threshold-simulating noise (TSN) to the model's internal representation of the signal. In this case, the TSN masks the inaudible information. This method is used, for example, in the BSIM and related models (Beutelmann & Brand, 2006; Beutelmann et al., 2010; Hauth et al., 2020; Roßbach et al., 2022).

In addition to reduced audibility, suprathreshold or cognitive processing deficits may be present, which may also be considered by a model, for instance, by using an individual suprathreshold component (ISC). However, one has to be aware that the manner of ISC implementation in a model does not necessarily reflect the underlying process in the real listener. In models, the ISC can be realized in several ways: Plomp (1978) proposed an ISC that depends on the broadband level of the masking noise and that is fitted to the listener's SRT in noise. This component has been termed distortion component (D-component) and can also be interpreted as a multiplicative noise (added to the input signal) or an additive noise after a (logarithmic) compression stage in the auditory pathway. This concept of a multiplicative noise is used, for example, by Hülsmeier et al. (2022) and Hülsmeier and Kollmeier (2022). Note that the D-component, according to the definition of Plomp (1978), does not necessarily reflect a non-linear distortion, as a frequency-dependent hearing loss can have a similar effect on the SRT.

Vicente et al. (2020), Vicente et al. (2021) and Lavandier et al. (2021) modified the binaural model of Lavandier et al. by simulating hearing loss using a level-dependent hearing threshold and by simulating suprathreshold processing deficits using an additional noise component. To do this, the hearing loss was divided into two parts that were roughly interpreted as estimates of the outer hair cell loss and the inner hair cell loss. The internal noise was composed of two components, one related to elevated thresholds and the other considering suprathreshold effects that depend on the level of the external noise. This approach predicted the HI listeners’ data but its parameters need to be fitted to the measured data. As far as we know, only hearing-impaired listeners with symmetrical hearing loss have been analyzed so far.

BSIM predicts the consequence of hearing loss using uncorrelated TSNs for the left and right ears without any suprathreshold component. This approach was used in previous studies to predict SRTs of hearing-impaired listeners for different spatial conditions in terms of reverberation of the room and positioning of the speech and noise (Beutelmann et al., 2010; Beutelmann & Brand, 2006). Beutelmann and Brand (2006) found a coefficient of determination (*R*^2^) of 0.83 for NH listener and 0.85 for HI between predicted and measured data. Beutelmann et al. (2010), using the same conditions, found an *R*^2^ of 0.87 for NH listener and 0.85 for HI listener. However, it is still unclear whether the observed effects can be attributed entirely to the peripheral hearing loss of the two ears (as in the case of BSIM modeled by the two TSNs) or whether BSIM would profit from including an ISC, since release from masking and/or speech recognition may be hampered more than the loss of audibility in the two ears would predict. We address this question in this study by applying BSIM with TSN, and, furthermore, by evaluating whether adding an ISC improves the accuracy of BSIM predictions. We include the ISC in two ways: firstly, by using individual SII references, and secondly, by employing an individually increased external noise for each listener (see below for details).

We analyzed SRTs of 738 NH and HI listeners with symmetrical, asymmetrical, and unilateral hearing loss in three different spatial conditions. Firstly, speech and noise were presented from the front (S_0_N_0_). Secondly, speech was presented from the front and noise was presented from either 90° or −90° azimuth, corresponding to the listener's worse ear (S_0_N_90_). And thirdly, speech and noise were spatially presented as in the S_0_N_90_ condition, but the signal was presented via the headphones solely to the better ear (BE) (S_0,m_N_90,m_). By taking the differences between the SRTs of these three conditions, it is possible to derive SRM, BEL, and BRM.

Note that our modeling approach does not aim to identify the precise cognitive or audiological limitations that may cause the ISC. Instead, our focus is on evaluating the overall impact of this ISC regardless of its origin. In short, this study addresses the following questions:

1. How well can BSIM predict SRTs for different listening conditions and how well can BEL, BRM, and SRM be predicted in HI listeners from their pure-tone audiograms alone?
2. Can the model's predictions be significantly improved by introducing an ISC? This is evaluated using two slightly differing manners of ISC.
3. Can we use significant deviations between predicted and measured SRTs to identify listeners with striking features in their auditory speech processing, thus identifying potential candidates for further audiological diagnostics?

## Methods

### Database

The Hörzentrum Oldenburg gGmbH provided a diagnostic database for this study. The data base is described in more detail in Afghah et al. (2023). The listeners came voluntarily to the Hörzentrum for special diagnostics. The database contains demographic information about the listeners and their health status, as well as the results of various audiological hearing tests. This data is available for 1,417 listeners (815 males and 602 females) with normal hearing, symmetrical hearing loss, asymmetrical hearing loss, and unilateral hearing loss. The age of the listeners ranged from 13 to 91 years (*M* = 64 years).

The SRTs were measured using the Göttingen sentence test (GÖSA; Kollmeier & Wesselkamp, 1997) presented via Sennheiser HDA 200 headphones. Three spatial conditions were realized by convolving the speech material and the interfering noise with head-related transfer functions (HRTFs; https://sound.media.mit.edu/resources/KEMAR.html). The interfering noise was the speech-simulating stationary noise of the Göttingen sentence test. The measurement involves three different spatial conditions: In all conditions the speech was presented from the front. The noise was presented once from the front (S_0_N_0_) and once from the 90° azimuth angle to the worse ear (S_0_N_90_). Additionally, the S_0_N_90_ condition was presented both binaurally and monaurally (that is, only the headphone channel of the BE was used). The worse ear was identified by medical anamnesis before the measurement. An illustration of the measurement setup is provided in the supplemental material. The interfering noise was spectrally matched to the long-term frequency spectrum of the sentences. One of 20 perceptually equivalent test lists consisting of 20 common sentences of three to seven words was used for each SRT measurement. Listeners were asked to repeat the presented sentence word by word, and the speech level was adjusted depending on the listeners’ responses according to an adaptive procedure that converged to a word recognition rate of 50%. Subsequently, a maximum likelihood estimator was used to fit the SRT (Brand & Kollmeier, 2002). The noise was presented at a fixed level of 65 dB SPL.

**Figure 1.**
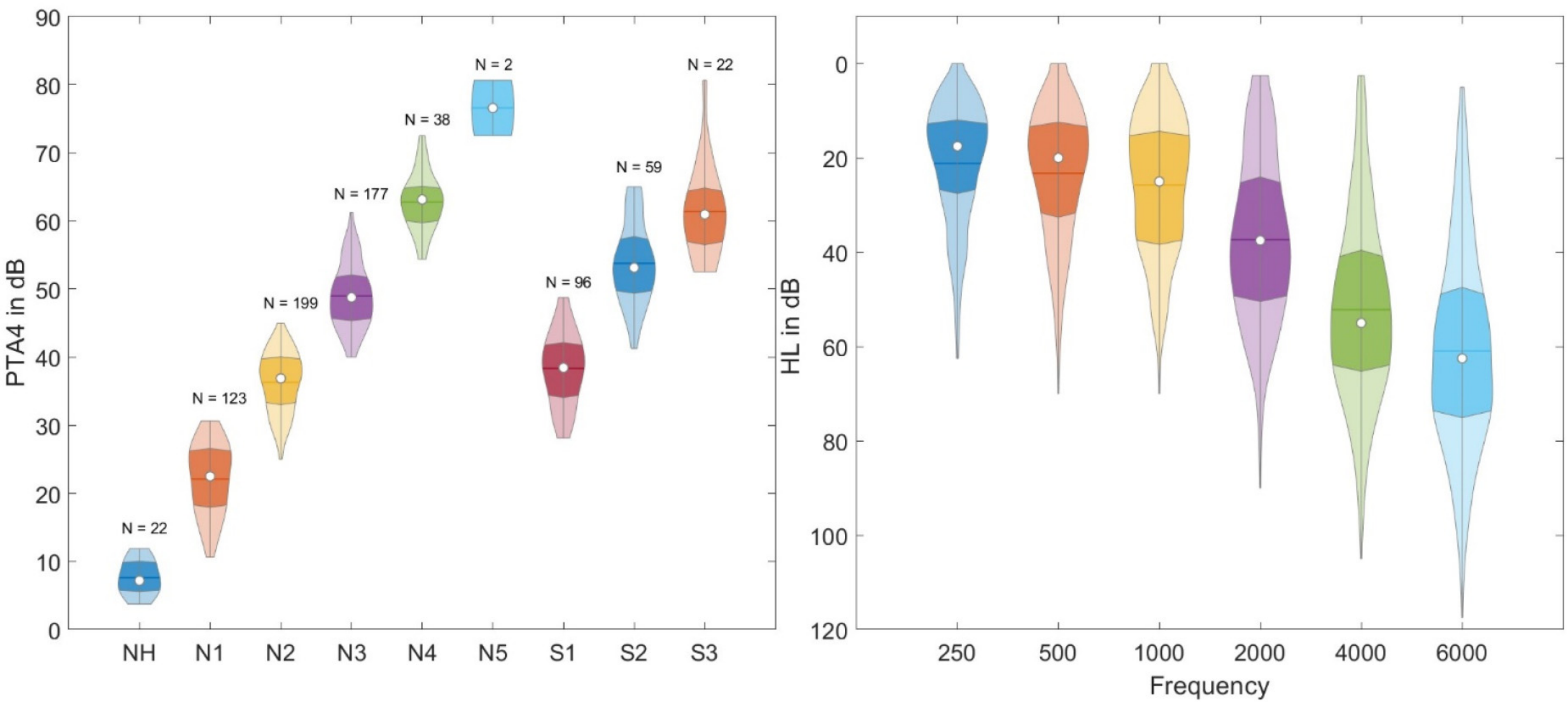
Overview of the hearing loss of the participating listeners. Left panel: violin plots depicting pure-tone average (PTA) in dB for NH listeners and HI listeners sorted into Bisgaard groups. Right panel: violin plots of the hearing loss of all listeners (averaged over both ears) in dB for different audiogram frequencies.

Our analysis only includes listeners for whom we had results for all spatial conditions and a complete pure-tone audiogram. In total, 738 listeners (292 females and 446 males) met these requirements. Figure 1 (left panel) shows the distribution of the pure-tone audiograms of these listeners sorted according to Bisgaard groups (Bisgaard et al., 2010). The Bisgaard groups are a classification system which is used to categorizes individuals based on the degree and type of hearing loss. It ranges from mild to profound hearing loss, with specific audiometric criteria defining each group. Furthermore, the S groups have a significantly steeper hearing loss across frequencies than the *N* groups. Figure 1 (right panel) shows the hearing loss averaged over both ears for audiogram frequencies ranging from 250 to 6000 Hz.

### Binaural Speech Intelligibility Model (BSIM)

#### Front-End: Blind Frequency Independent EC Processing

The Binaural Speech Intelligibility model (BSIM) in the version of Hauth et al. (2020) consists of a blind EC mechanism (see below) as the front-end and the non-blind SII according to ANSI (S3.5-1997) as the back-end. The front-end is blind, as it only gets the mixed speech and noise signals for the left and right ears without any further auxiliary information about these signals. The listener's hearing loss is realized by adding two TSNs to the left and right input signals. The left and right TSNs are generated as uncorrelated signals, so that the following EC stage cannot cancel them out. This TSN was also used in previous BSIM versions by Beutelmann and Brand (2006) and Beutelmann et al. (2010). The frequency selectivity of the auditory system is simulated with a gammatone filter bank (Hohmann, 2002) dividing the input signal into 30 equivalent rectangular bandwidth (ERB) spaced frequency bands (Moore & Glasberg, 1983). In frequency channels above 1,500 Hz, BSIM selects the BE. In each frequency channel below 1,500 Hz, BSIM applies an independent EC processing as follows: In the equalization step, the left and right input signals are equalized in level and phase, and in the cancellation step, two concurrent strategies are applied to improve the SNR. Firstly, the signals are subtracted from each other, which is a useful strategy at negative SNRs, because the noise is cancelled causing a minimization of the model's output level. Secondly, the signals are added to each other, which is a useful strategy at positive SNRs, because the speech signal is increased, causing a maximization of the model's output level.

The EC strategy (minimization or maximization) at low frequencies and the BE (left or right) at high frequencies is chosen blindly using the speech-to-reverberation modulation energy ratio (SRMR) in each of the channels independently (Santos et al., 2014). The SRMR is defined as the ratio of the power in the four lowest modulation filters to the power in the four highest modulation filters. This ratio indicates how speech-like the signal is with respect to this simple modulation analysis. The SRMR is generated using the results of both the minimizing and maximizing EC pathways for frequency channels up to 1500 Hz. For subsequent processing, the strategy that produces the greater SRMR score is chosen in each channel. This EC process aims at modeling human binaural processing performance and is therefore not supposed to work perfectly. Therefore, uncertainties have been implemented via a jitter applied directly to the signal. The jitter varies in delay and gain factors within the EC process. To model these variations, we employ Monte Carlo simulations (MCSs), where the deviations from the optimal values (as determined through the minimization or maximization of the EC output), are sampled from normal distributions with standard deviations according to Vom Hövel (1984; see also Beutelmann & Brand, 2006). The outputs of all gammatone channels (which either went through the EC processing or were selected according to the BEL) can then be combined to produce a single-channel signal, which can be analyzed by arbitrary speech intelligibility models. A visual representation of the model is provided in the supplemental material (see Figure S2 in the supplemental material).

#### Back-End: Speech Intelligibility Index (SII)

As all processing steps of the model front-end are linear, it is possible to store its settings used for the mixed speech and noise signals and to apply this processing to the speech and noise separately. Mixing these separately processed signals after the processing results in an identical signal as the output of BSIM. This approach can be regarded as an ideal “shadow filtering,” as proposed by Hagerman and Olofsson (2004) and it enables the application of non-blind back-ends. In this study, we used the non-blind SII as follows: The SNR is calculated for the 30 output gammatone bands of the front-end, normalized, weighted according to the SII band importance function, and summed up across all bands. In contrast to the SII standard, no hearing loss is considered in the SII calculation, as the hearing loss has already been considered by the TSN that was added to the input signals in the front-end. Note that the example SII procedure described in ANSI S3.5-1997 proposes different filter banks than the gammatone filter bank. Nevertheless, the standard describes how alternative filter banks can be applied. We followed this recommendation when using the gammatone filter bank. The result is an index value between 0 and 1, where 1 indicates the highest level of intelligibility. The mapping of this index to the empirical data is described in the next section.

#### SRT Calculation

For each SRT prediction, 10 sentences (consisting each word of the speech material once) were used to ensure consistent predictions regardless of the chosen sentences. Each sentence was processed with 10 Monte-Carlo simulations of the internal processing inaccuracies, as described in the previous section (resulting in 100 simulations per SRT in total). This was done for SNRs between −20 and 20 dB in one-dB steps. Given that the SII does not inherently provide information about speech intelligibility, we mapped the predicted SII values to SRT. We employed a single reference index value (0.2) for all listeners and spatial conditions when we used no ISC. This general reference SII value of 0.2 was chosen because it was used in previous studies in which BSIM was applied to the Göttingen sentences.

In order to investigate the potential improvement of the predictions, we introduced an ISC in two distinct ways: Firstly, we used an individual reference SII value for each listener, i.e., the SII value that corresponds to the SRT of this listener in the S_0_N_0_ condition. We used the SII value corresponding to the measured SRT of the S_0_N_0_ condition to determine the reference SII and used this for predicting SRTs for the S_0_N_90_ and S_0,m_N_90,m_ conditions for each listener. Secondly, we used an alternative method by increasing the external noise level by the difference between the measured S_0_N_0_ and the predicted S_0_N_0_. In this way, we consider that speech understanding of the individual HI listener might be disturbed more severely by the external noise than predicted by the standard model. When the SRT is overestimated (in the S_0_N_0_ condition), it means that the participants showed better intelligibility than predicted, which might be due to some ability of the listener to compensate for the individual hearing loss. The increased external noise method does not consider such compensation effects. The reference SII used, was always 0.2, as with the calculation without ISC.

In summary, this means that we have three different methods for calculating and comparing SRTs:

1. A common reference value for all participants (no individualization)
2. Individual SII reference value for each listener (ISC_SII_)
3. Individual increased external noise for each listener (ISC_Noise_).

Note that the difference between the two ISC adjustments is that adapting the reference SII can shift the predicted SRTs towards either higher or lower values, while the individual increased external noise can only shift the predicted SRTs towards higher values. A visual representation of the model and the implemented ISCs is provided in the supplemental material (see Figure S2 in the supplemental material).

The comparison of the S_0_N_0_, S_0_N_90_, and S_0,m_N_90,m_ results allowed us to calculate SRM, BRM, and BEL.

1. Spatial Release from Masking (SRM)** **=** **S_0_N_0_–S_0_N_90_:
  In the S_0_N_0_ condition, no spatial separation between speech and noise is possible as both signals come from the same direction. This leads to no BEL and no binaural benefits, due to no differences between the two ears. In contrast, the S_0_N_90_ condition leads to BEL, because one ear has a better SNR. Furthermore, there is a BRM due to the temporal differences between the ears. Consequently, calculating the difference between SRTs of the S_0_N_0_ and the S_0_N_90_ conditions reveals SRM, which is the combined effect of BEL and BRM.
2. Binaural Release from Masking (BRM)** **= S_0,m_N_90,m_–S_0_N_90_:
  In the S_0,m_N_90,m_ condition the speech and noise signals are presented monaurally to the ear with the better SNR. If we now switch on the ear with the worse SNR (S_0_N_90_ condition), no BEL effect is present and the measured benefit can only be due to binaural processing. Consequently, calculating the difference between S_0,m_N_90,m_ and S_0_N_90_ isolates the binaural benefit.
3. Better Ear Listening (BEL)** **= S_0_N_0_–S_0,m_N_90,m_:
  As mentioned above, neither BEL nor binaural benefit are present in the S_0_N_0_ condition, while only BEL is present in the S_0,m_N_90,m_ condition. Consequently, calculating the difference between SRTs of the S_0_N_0_ and S_0,m_N_90,m_ conditions isolates the BEL effect.

#### Statistical Method

In this study, we calculate, for every spatial condition and SRT prediction method, the coefficient of determination (*R*^2^), the prediction error (bias), and the root-mean-squared error (RMSE). *R*^2^ indicates the percentage of the measured SRT variance that the model can explain. The bias indicates the systematic error between predicted and measured SRTs. The RMSE indicates the standard deviation between predicted and measured data. We used Fisher's *Z*-transformation to assess whether the coefficients of determination found for the different listening conditions and prediction methods differed significantly.

## Results and Discussion

**Figure 2.**
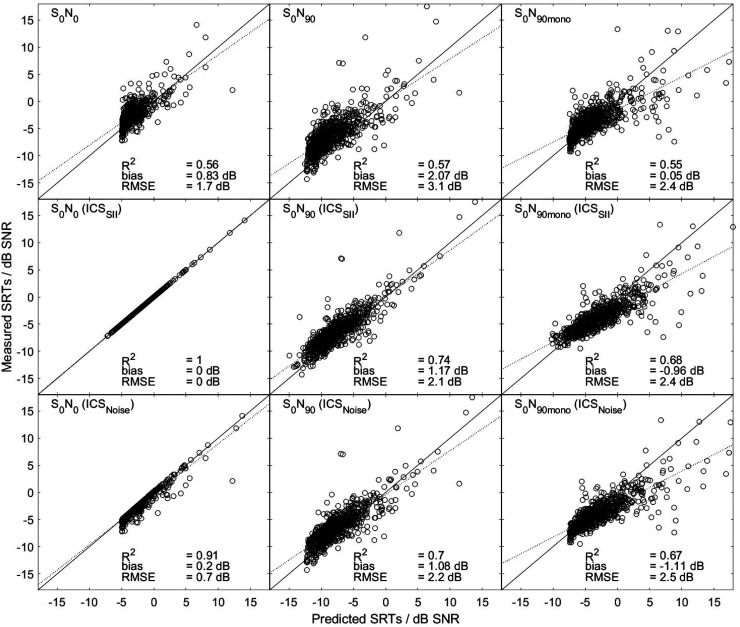
Relationship between predicted and measured SRTs for the three spatial conditions using BSIM across three different SII references: in the top row one common SII reference (no ISC) was used for all listeners. In the middle row an individual SII reference (ICS_SII_) was used for each listener matched to the S_0_N_0_ condition. In the bottom row an individually increased external noise (ISC_Noise_) component matched to the measured S_0_N_0_ was employed for each listener. Each row shows three spatial conditions: S_0_N_0_ condition (left panel), S_0_N_90_ condition in dichotic presentation (middle panel), and S_0,m_N_90,m_ condition in monaural presentation utilizing the listener's better ear (right panel). Each panel includes the coefficient of determination (*R*^2^), bias, and root mean square error (RMSE). The solid black line represents perfect agreement, while the black dotted line is a linear regression line fitted to the data.

Figure 2 shows scatter plots representing measured SRTs (*y*-axis) against predicted SRTs (*x*-axis) for all 738 subjects. The left panels show the results for the S_0_N_0_ condition, the middle panels show the S_0_N_90_, and the right panels show the S_0,m_N_90,m_ condition. In all panels, we include *R*^2^, prediction bias, and RMSE. The three rows in the plot differ in the individualization of the SRT prediction. The top row shows the predictions using a common SII reference value for all listeners; no individualization was carried out. The *R*^2^ between predicted and measured SRTs were *R*^2^ = 0.56 (S_0_N_0_), *R*^2^ = 0.57 (S_0_N_90_), and *R*^2^ = 0.55 (S_0,m_N_90,m_). In this condition, a noteworthy observation is the positive correlation between predicted and observed SRTs and the increasing variance of predicted SRTs with increasing SRT values. The middle row shows the predictions using ISC_SII_, i.e., using an individual SII reference value for each listener. This individual reference was set to match the observed value for the S_0_N_0_ condition. Consequently, the SRTs for the S_0_N_0_ condition (left panel) were not predicted, but fitted. For the S_0_N_90_ and the S_0,m_N_90,m_ conditions, the use of the individual SII reference improved the squared correlation coefficients to *R*^2^ = 0.74 (S_0_N_90_) and *R*^2^ = 0.68 (S_0,m_N_90,m_) compared to the predictions using a common reference value shown in the top row. A subsequent Fisher’s test confirmed that this improvement was significant (see Table S1 in the supplemental material for statistical details). The RMSE decreased from 3.1 dB (common reference) to 2.1 dB in the S_0_N_90_ condition but did not differ for the S_0,m_N_90,m_ condition.

The bottom row shows the predictions using ISC_Noise_, that is, using an individually increased external noise for each listener. In both spatial conditions (S_0_N_90_ and S_0,m_N_90,m_), the *R*^2^ was enhanced to 0.7. Here, too, a subsequent Fisher's test confirmed that this improvement was significant as opposed to the model predictions without individualization. Note that also here the SRTs in the S_0_N_0_ condition were not predicted, but rather serve to determine the listeners’ increased external noise levels. Using ISC_Noise_ led to a decrease in RMSE from 3.1 to 2.2 dB for the S_0_N_90_ condition and to an increase in RMSE from 2.4 to 2.5 dB for the S_0,m_N_90,m_ condition.

Fischer's test of the differences between *R*^2^ values revealed no statistically significant difference between the two ISC methods.

**Figure 3.**
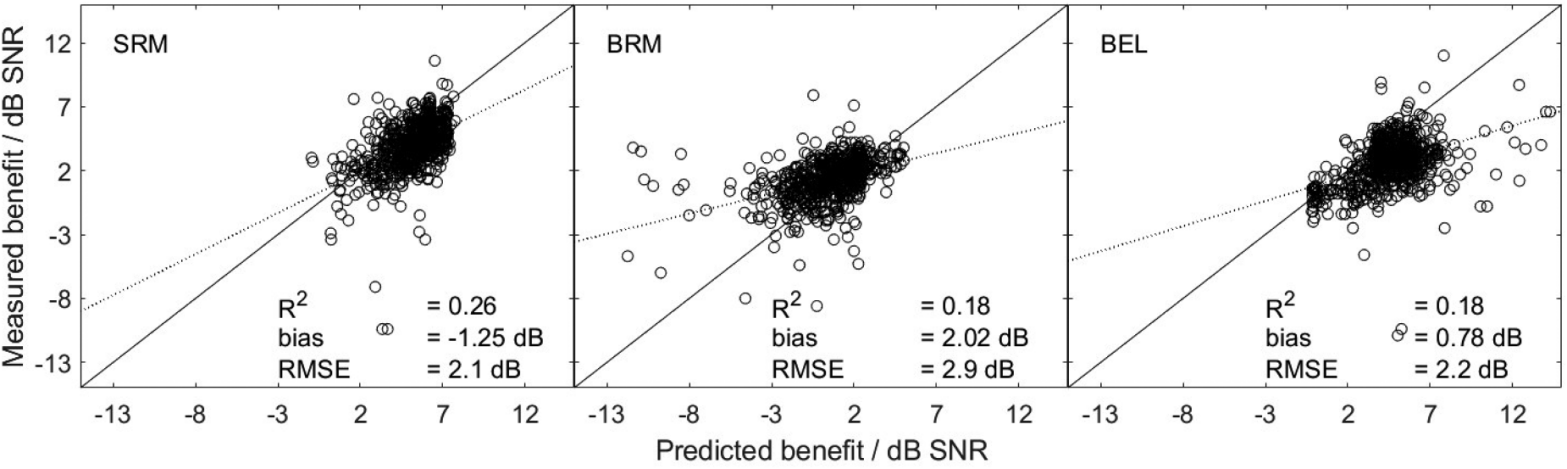
Measured vs. predicted SRM (S_0_N_0_–S_0_N_90_, left panel), BRM (S_0,m_N_90,m_–S_0_N_90_, right panel), and BEL (S_0_N_0_–S_0,m_N_90,m_, right panel). Each panel includes the coefficient of determination (*R*^2^), bias, and root mean square error (RMSE).

Figure 3 shows the measured versus the predicted SRM (S_0_N_0_–S_0_N_90_, left panel), BRM (S_0,m_N_90,m_ –S_0_N_90_, middle panel), and BEL (S_0_N_0_–S_0,m_N_90,m_, right panel). In all cases, *R*^2^ was low, with 0.26 for SRM and 0.18 for BRM and BEL. All correlations (SRM: *R* = 0.51, *p* = 2.2 × 10^−16^, confidence range = [0.45, 0.55]; BRM and BEL: *R* = 0.36, *p* = 2.2 × 10^−16^, confidence range = [0.36, 0.48]) were significant. The predictions regarding spatial and binaural benefits resulted in a notably low *R*^2^. This can, in part, be attributed to the limited variance in the measured data (standard deviation (*SD*) of SRM: *SD* = 2.1 dB; BRM: *SD* = 3.3 dB; BEL: *SD* = 3.0 dB). Note that the use of an ISC does not affect the prediction of SRM, BRM, or BEL, as these measures are based on differences in SRTs and thus the ISC is compensated in the calculation.

**Figure 4.**
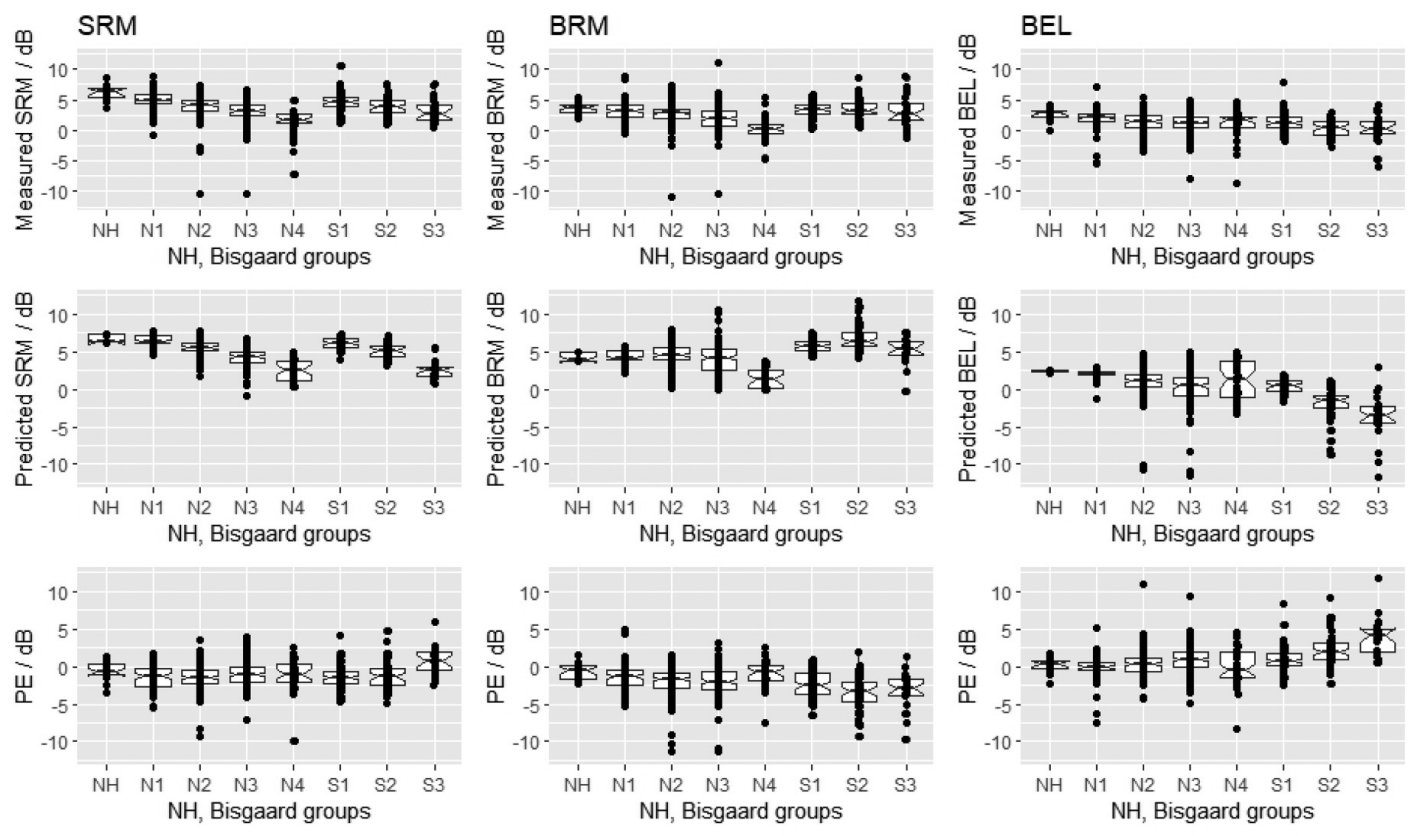
Comparison of the measured (upper panels) SRM (left), BRM (middle), and BEL (right), the predicted data with the BSIM model (middle panels) and the prediction error between the measured and predicted data (lower panels). All results are sorted into NH and Bisgaard groups. Note: Because there were only two participants in the Bisgaard group N5, we did not include this group in the analysis.

Figure 4 shows boxplots of the measured SRM, BRM, and BEL in the upper panels, predicted SRM, BRM, and BEL in the middle panels and the prediction error (measured minus predicted SRTs) of SRM, BRM and BEL in the lower panels for NH listeners and the Bisgaard groups. The left panels show the SRM, the middle panels show BRM, and the right panels show BEL.

There were significant differences in measured SRM and BEL between almost all groups. This was not the case for BRM. Here, only groups N3 and N4 differed significantly from all other groups. These two groups had a larger hearing loss in low frequencies than the other groups. This frequency range is most important for BRM, as time and phase differences can only be analyzed by human listeners at low frequencies according to the duplex theory (Rayleigh, 1907). We set the transition between low and high frequencies to 1.5 kHz in our model. For more details, see Tables S4 to S15 in the supplemental material.

For the predicted SRM and BEL there were also significant differences between almost all groups. For the predicted BRM, a significant difference was found for the N4, S1, S2, and S3 groups. In all other groups and predictions, no consistent characteristics were observed across the results.

A further goal of this study was to test whether deviations between predictions and measurements for individual listeners may help to identify outliers with striking auditory or cognitive features. We used the Kolmogorov–Smirnov (KS) test to analyze the distribution of prediction errors and found a significant deviation from a normal distribution. In order to test whether this deviation was caused by outliers, we identified outliers using the interquartile range (IQR) method. The IQR is calculated as the difference between the third quartile (Q3) and the first quartile (Q1) of the data, expressed mathematically as the difference of Q3 and Q1. A common threshold for identifying outliers is 1.5 times the IQR. Data points falling below Q1 − 1.5 × IQR or above Q3 + 1.5 × IQR are considered potential outliers (Tukey's fences method). After 7 identified outliers were excluded, the KS test found no significant deviation of the remaining data from a normal distribution and, consequently, we could not separate specific listeners with striking results in the remaining distribution (for more information, see Table S2 in the supplemental material). An analysis of the outliers with respect to 22 parameters provided in the data base, such as age, PTA4, hearing loss, predicted SRTs, tinnitus, and cognitive tests did not reveal any systematic or striking deviations for these outliers.

In addition, we analyzed the measured and predicted values of SRM, BRM, and BEL using one-sided pairwise comparisons between the different Bisgaard groups, as shown in Table 1. We have developed a representation that visually illustrates the relationships between the predicted data and the measured data for the different hearing loss groups and termed it *pattern projection table*. The idea behind this representation is to show which hearing loss groups differ significantly in the measured data and whether the model can predict these differences. A pattern therefore emerges from the measured data and can be compared with the pattern of the model. The pattern projection tables (Table 1) display the results for SRM in the left panel, BRM in the middle panel, and BEL in the right panel. The crossed-out area indicates comparisons that would be redundant. Each panel shows the results of two one-sided pairwise comparisons for the measured and predicted data.

**Table 1.** Pattern Projection Table Showing Pairwise Comparisons Between Bisgaard Groups Based on Measured Data and Predictions.

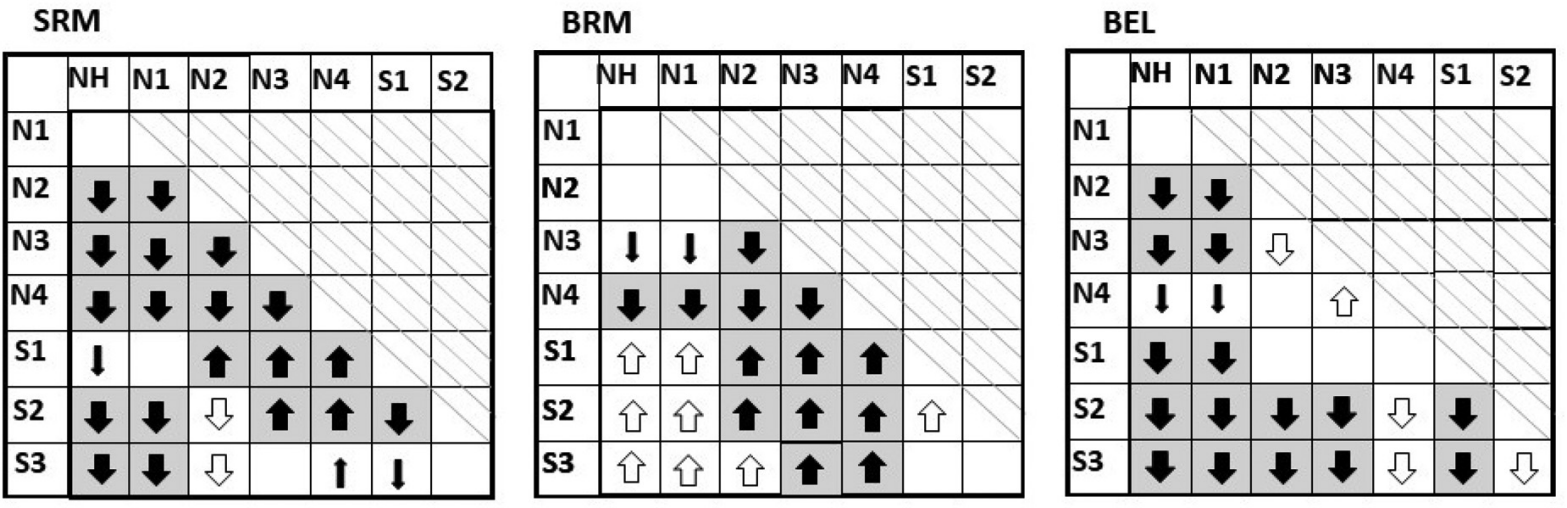

The large solid arrows with a gray background indicate that both the measured and predicted data show significant differences between two groups. These significant differences between two groups only occurred in the same direction. The small filled arrows indicate that only the measured data showed a significant difference between two groups and the predictions did not. The open arrows indicate that only the predicted data showed a significant difference between two groups and the measurements did not. Empty fields indicate that neither measurements nor predictions found a significant difference. The direction of the arrows indicates which of the two possible hypotheses was found (for more details see text below).

**Table 2.** Comparison of the Effects of Introducing the ISC_SII_ Between the non-Blind (Upper Rows) and Blind EC Stage (Lower Rows).

Model	Condition	Metric	S_0_N_0_	S_0_N_90_	S_0,m_N_90,m_	SRM	BRM	BEL
BSIM10	no ISC	*R* ^2^	0.58	0.6	0.57	0.3	0.25	0.11
		bias (dB)	1.89	4.01	3.07	−2.12	−0.94	−1.18
		RMSE (dB)	2.4	4.6	3.7	2.8	1.9	2.1
	ISC_SII_	*R* ^2^	1	0.74	0.67			
		bias (dB)	0	2.01	1.16			
		RMSE (dB)	0	2.7	2.1			
BSIM	no ISC	*R* ^2^	0.56	0.57	0.55	0.26	0.18	0.18
		bias (dB)	0.83	2.07	0.05	−1.25	−2.02	0.78
		RMSE (dB)	1.7	3.1	2.4	2.1	2.9	2.2
	ISC_SII_	*R* ^2^	1	0.74	0.68			
		bias (dB)	0	1.17	−0.96			
		RMSE (dB)	0	2.1	2.4			

During these comparisons, two hypotheses were tested:

1. The mean of the first group (indicated by the table column) is greater than the mean of the second group (indicated by the table row).
2. The mean of the first group is smaller than the mean of the second group.

The order of the hearing loss groups is based on the severity of the hearing loss and can be recognized by the horizontal and vertical designations in Table 1. This means that when two groups are compared in pairs, the first group is always the one with the lower hearing loss. The direction of the arrows indicates which of the two hypotheses were fulfilled. An upward arrow indicates that hypothesis one was met, while a downward arrow indicates that the second hypothesis was met. The large solid arrow with a gray background indicates that the significant difference was found for both the measured and predicted data. If the difference was only found in the measured data and not in the predictions (type II error), it is marked with a small filled arrow in the table. If a difference was found only in the predictions and not in the measurements (type I error), it is marked with an open arrow. These significant differences between two groups only occurred in the same direction. Of all 63 possible pair comparisons, 47 pair comparisons (74%) of the model matched the measured data. The average effects were predicted fairly well, but not in all cases. The predictions for the SRM were very close to the measured data, with the exception of a few cases. With the BRM, the significant differences in the S groups were only predicted by the model, but no significant difference was found in the measured data (type I error). For the BEL, most of the pairwise comparisons of the data and the model were consistent.

After showing that the ISC yielded higher prediction accuracy in combination with the blind EC stage, we compared the results with a previous version of BSIM with a non-blind EC stage (see Table 2).

The results show that BSIM10 yields slightly higher *R*^2^ for S_0_N_0_, S_0_N_90_, and S_0,m_N_90,m_, and SRM conditions in the comparison without an ISC, but the current model yields lower bias and lower RMSE. For the BRM condition, BSIM10 yields lower bias and lower RMSE, while for BEL the current model yields higher *R*^2^. In summary, BSIM10 achieves slightly higher *R*^2^ values only for the predictions without ISC, while the current model generally results in lower bias and RMSE for all prediction, with exception depending on the specific condition. For the predictions with the ISC_SII_, both models achieve the same *R*^2^. For S_0_N_90_, the current model still yields lower bias and RMSE than BSIM10, but this was not the case for S_0,m_N_90,m_. As mentioned before, the ISC_SII_ has no influence of SRM, BRM, and BEL.

## General Discussion

One objective of this study was to evaluate BSIM for a large number of hearing-impaired individuals. Our *R*^2^ and RMSE findings (S_0_N_0_: *R*^2^ = 0.56, RMSE = 3.1 dB; S_0_N_90_: *R*^2^ = 0.57, RMSE = 3.1; S_0,m_N_90,m_: *R*^2^ = 0.55, RMSE = 2.3 dB) confirm our expectation that BSIM can predict the SRTs of hearing-impaired listeners to a certain degree. In Beutelmann et al. (2010) the conditions from Beutelmann and Brand (2006) were predicted again using the model of Beutelmann et al. (2010). For a better readability, we refer to the models as BSIM06 and BSIM10. Using BSIM06 and BSIM10 resulted in much better prediction accuracy between measured and predicted SRTs (BSIM06: *R*^2^ = 0.91; BSIM10: *R*^2^ = 0.92) across different reverberant rooms and azimuth angles of the noise and listeners with and without hearing loss. This previous models used the listeners’ audiograms, only to address the hearing loss, as in the current study without ISC. The superior prediction accuracy of BSIM06 and BSIM10 is probably caused to a certain degree by the variance of the measured data, as they varied the azimuth of the interfering noise, whereas the current study used a constant noise azimuth; the entire variance analyzed in this study arise from interindividual differences. Furthermore, the better prediction accuracy may have arisen from the relative simplicity of the non-blind front-end of BSIM06 and BSIM10. The blind front-end in the current model does not receive any information about which parts of the input signal are speech and which are noise. BSIM10 calculates the binaural benefit (as well as the consequence of interaural processing inaccuracies) analytically under the assumption that the optimal values of the interaural equalization parameters can be found even though the speech and noise signals may be completely inaudible. This differs from the current model with the blind front-end, where the raised threshold simulating noise already affects the binaural processing, resulting in less optimal performance, which we regard as more realistic.

The current study explores a much more diverse group of listeners than Beutelmann and Brand (2006) who used 15 participants, while the current study used 738 participants. A further explanation for the differences between studies may be found in the speech material and the training state of the participants. The participants in Beutelmann and Brand (2006) underwent a training session of two sentence lists with 20 sentences each from the Oldenburg sentence test, and then listened to 1 set of 20 sentences for the testing, whereas the listeners in the current study received no training and listened to 3 sets of 20 sentences from the Göttingen sentence test, which are known to be somewhat less reproducible than the Oldenburg sentences (Brand & Kollmeier, 2002). The comparison shows that both models yield higher prediction accuracy with introducing the ISC_SII_. Furthermore, the results show, that BSIM yield higher predictions accuracy despite the fact, that the model requires less information.

A further objective of this study was to test whether an ISC can be used to improve the model predictions (see Figure 2). We used two methods to realize an ISC: an individual SII reference value and an individually increased external noise. The main difference between these two methods is that predicted SRTs can either increase or decrease when using an individual SII reference value, whereas they can only increase when using the individually increased external noise. Both approaches resulted in significant improvements in prediction accuracy for the S_0_N_90_ and S_0,m_N_90,m_ conditions compared to using no ISC. No significant difference was observed between the improvements introduced by the two methods. The introduction of a distortion component was also examined in a study by Hülsmeier et al. (2022). They used a clinical dataset with SRTs of 315 ears (Wardenga et al., 2015). That study addressed speech intelligibility predictions using three different models: framework for auditory discrimination experiments (FADE), SII, and PAV, with PAV denoting an adjusted SII with a hearing loss-dependent band importance function, introduced by Pavlovic et al. (1986). The prediction errors of the models based on the attenuation component were used to implement the distortion component. After this adjustment, the models achieved the following RMSE values: 3.8 dB (6.5 dB before adjustment) for FADE, 7.1 dB (6.8 dB before adjustment) for SII, and 3 dB (6.3 dB before adjustment) for PAV. These values are comparable to the RMSEs found in the current study. In both studies, the measured variance is caused solely by interindividual differences between listeners.

The binaural model of Vicente et al. (2020) also uses the listener's pure-tone audiogram and an additional component based on the level of the external stimuli. Their study included three different headphone experiments, where two anechoic noise vocoded speech maskers were presented either from the same direction as the target or spatially separated from the target. The experiments investigated the effects of spatial conditions, type of noise masker, and audibility on measured and predicted speech intelligibility. This approach achieved an overall accuracy measured by the Pearson's correlation coefficient of 0.93 or more. The mean absolute prediction error was 1.1 dB or smaller. The maximal absolute error was 3.1 dB. Note that these results refer to the mean of all results, so the interindividual deviation was not considered as it was in the present study. Furthermore, Vicente et al. (2020) used data from 10 NH and 10–13 HI listeners drawn from different pools of participants across three experiments. Moreover, they included only HI listeners with a sensorineural hearing loss and a maximal difference of 10 dB HL between the left and right ears, which is a more consistent sample of listeners than used in the current study.

In addition, the analysis can go a little further. For instance, Vicente et al. (2020) fits the parameters of their internal noise to obtain the most accurate predictions on average across conditions and listeners of the two hearing groups (HI and NH). The model proposed by Lavandier et al. (2021) included an additional parameter that must be fitted, but the value was the same for any participant. This is a different approach as opposed to the one proposed in the current study, for which we decided to implement the ISC_Noise_.

## Limitations of This Study and Outlook

In this study, the ISC is based on the SRT in the S_0_N_0_ condition. This has the disadvantage that the model no longer makes a prediction for the S_0_N_0_ condition. To avoid this, we analyzed whether it is possible to predict the ISC based on the hearing loss, as done by Hülsmeier et al. (2022). However, the prediction errors showed no significant dependence on the hearing loss (as specified by the Bisgaard group, see Figure 4). This holds for the S_0_N_0_, S_0_N_90_, and S_0,m_N_90,m_ conditions.

Note that our modeling approach is an extreme simplification of the complex perceptual effects of hearing loss. The relationship between audibility and suprathreshold effects on the one hand, and speech perception on the other, is influenced by many factors, including inner and outer hair cell loss, auditory neuropathy, cognitive abilities, and the use of speech context. We found that introducing a suprathreshold component leads to an improvement in prediction accuracy. This means that some of the participants benefited from the inclusion of this component. However, from the predictions alone, we cannot determine whether this arises from cognitive or auditory factors: our model does not reveal these relationships. Thus, this model's predictions are not suitable for drawing specific diagnostic conclusions for individual listeners. Nonetheless, our comparison of BSIM predictions with HI data highlights the possibilities and limitations of this predictive model and its potential contribution for characterizing the consequence of hearing loss on spatial and binaural unmasking of speech.

As the lower three panels in Figure 4 show, there is a remaining bias of the predicted BEL and BRM for the Bisgaard groups S2 and S3, that is, for HI participants with a larger high frequency loss. It may be possible to reduce this bias by a refinement of the model, for instance, by modifying the frequency weighting in the SII. However, we decided against refining the model at present because the mismatch is small and we see an advantage in evaluating the predictive power of existing model with this large data set.

## Conclusion

In this study, we modeled individual SRTs for NH and HI listeners using BSIM, as introduced by Hauth et al. (2020), in different spatial conditions. We tested whether the inclusion of an ISC characterizing the listener, in addition to his or her pure-tone audiogram, improves the prediction accuracy of the model. We also analyzed the binaural processing and spatial effects by separating SRM, BRM, and BEL. This study showed that BSIM can predict the individual SRT data of HI listeners even when using solely the listener's pure-tone audiograms. However, introducing an ISC results in a significant improvement in prediction accuracy. This can be realized by using either an individual SII reference value or individually increased external noise. Note that the predictions of SRM, BRM, and BEL are not influenced by the addition of an ISC, as these measures are based on differences between the SRTs in different listening conditions, so that the ISC is compensated in the calculation. The results show no correlation between the prediction error and the degree of hearing loss, as characterized by the grouping of hearing loss according to Bisgaard et al. (2010).

## Supplemental Material

sj-docx-1-tia-10.1177_23312165261418655 - Supplemental material for Analysis of Spatial, Binaural, and Better-Ear Benefits for Different Degrees of Hearing Loss Using a Binaural Speech Intelligibility Model Supplemental material, sj-docx-1-tia-10.1177_23312165261418655 for Analysis of Spatial, Binaural, and Better-Ear Benefits for Different Degrees of Hearing Loss Using a Binaural Speech Intelligibility Model by Saskia Röttges, Christopher Hauth, Kirsten C. Wagener and Thomas Brand in Trends in Hearing
