# Supplementary material for: Analysis of Spatial, Binaural, and Better-Ear Benefits for Different Degrees of Hearing Loss Using a Binaural Speech Intelligibility Model
Source: Trends Hear. 2026 Feb 12;30:23312165261418655. doi: 10.1177/23312165261418655 (PMC12901929; doi:10.1177/23312165261418655)
Supplement: sj-docx-1-tia-10.1177_23312165261418655 - Supplemental material for Analysis of Spatial, Binaural, and Better-Ear Benefits for Different Degrees of Hearing Loss Using a Binaural Speech Intelligibility Model [file sj-docx-1-tia-10.1177_23312165261418655.docx]

SUPPLEMENTAL MATERIAL


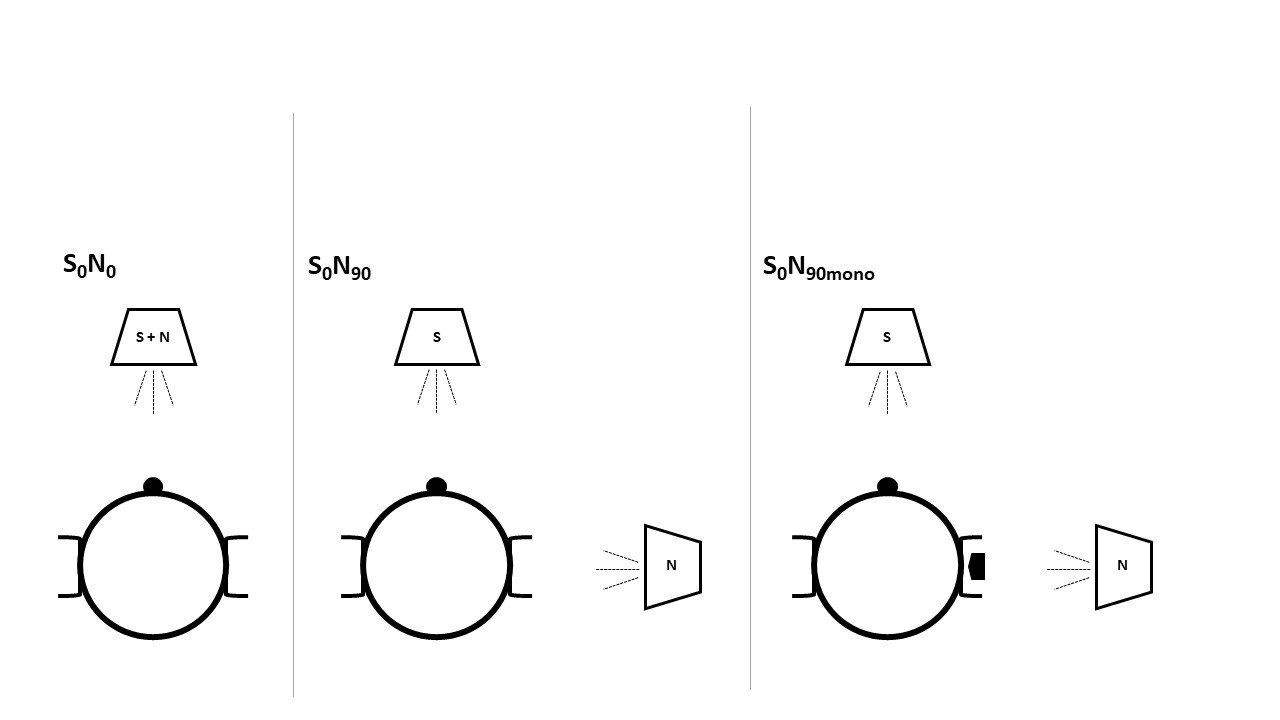


Figure S1: Illustration of the three measurement conditions. Speech was always presented frontally, and noise was presented from the front (S_0_N_0_, left panel) and from 90° azimuth to the worse ear (S_0_N9_0_). The S_0_N_90_ condition was performed both binaurally (S_0_N_90_, middle panel) and monaurally (S_0,m_N_90,m_, right panel), where only the better ear was addressed using only one channel of the binaural HRTF.


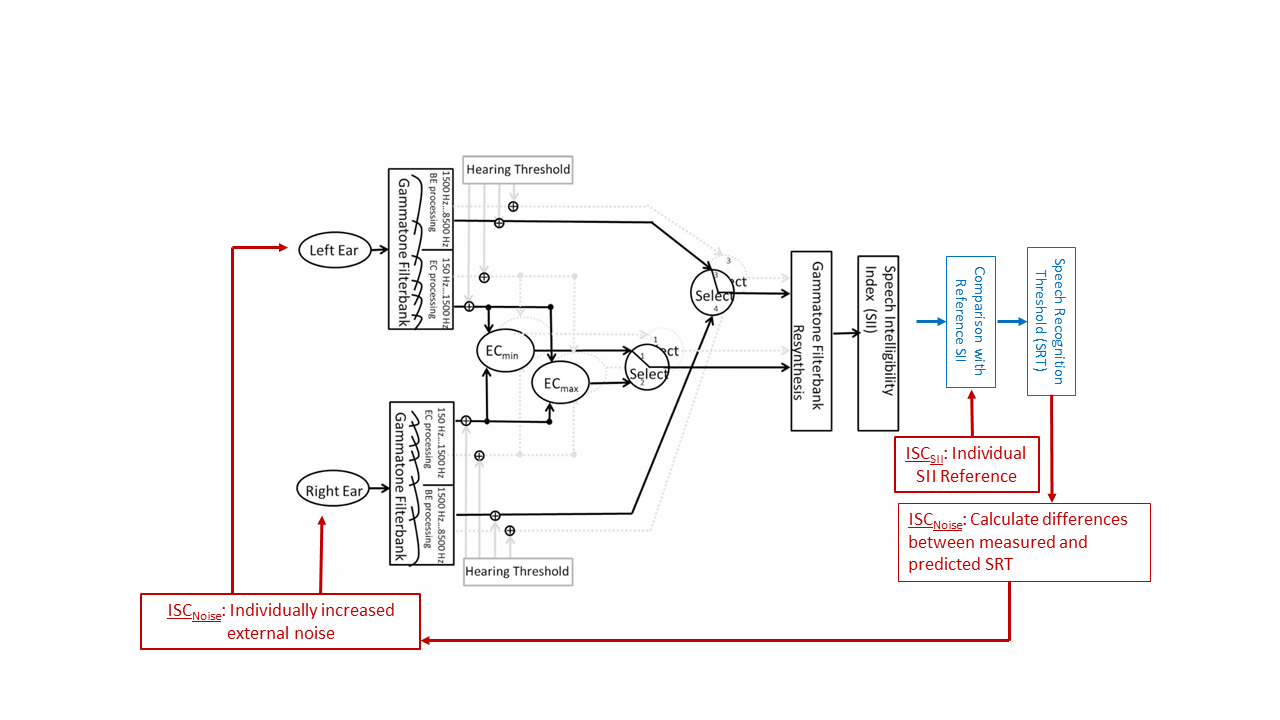


Figure S2: The black part of this sketch is the model introduced by Hauth et. al 2020: Block Diagram of the General Processing Performed in BSIM 2020. The mixed signals on the left and right ear are divided into 30 frequency bands ranging from 150 Hz to 8500 Hz using a gammatone filter bank (Hohmann, 2002). Afterward, frequency bands below 1500 Hz are fed to the EC stage, where both a level minimization and level maximization is performed in parallel, denoted as EC_min and EC_max. The speech-to-reverberation modulation ratio (SRMR; Santos et al., 2014), denoted as select-stage is used (a) for selecting if the level-minimization or the level-maximization produces the best SNR improvement and (b) for determining the better ear. This is indicated by the numbers in the selection stage. For low frequencies, either the EC-Min (1) or the EC-Max (2) path is selected. For high frequencies (above 1500 Hz), either the left ear channel (3) or the right ear channel (4) is selected. Both, binaurally processed channels and the better ear channels are combined, and a single-channel output is resynthesized using a gammatone synthesis filter bank. The output can then be analyzed by an arbitrary back end, which is the SII in this study. EC : equalization cancellation; SII : speech intelligibility index. The blue part illustrates the steps to calculate the SRT from the model’s output. The red part shows the introduced individual suprathreshold component (ISC) to improve the prediction accuracy in two ways. First, by using an individual reference SII value for each listener corresponding to their measured SRT in S_0_N_0_ condition to predict S_0_N_90_ and S_0,m_N_90,m_ conditions. Second, by individually increasing the external noise level by the difference between measured and predicted SRT in S_0_N_0_ condition.

TABLE S1: p values of Fisher’s test, in which the correlations of the three different methods of SRT calculation were compared: 1) common reference (no ISC), 2) individual SII reference (ISC_SII_), 3) individually increased external noise (ISC_Noise_). The original significance level (0.05) was adjusted using a Bonferroni correction, resulting in a corrected significance level of 0.016. Significant results in the table are indicated by stars.

| **Manner of individualization** | **Spatial condition** | |
| --- | --- | --- |
|  | S_0_N_90_ | S_0,m_N_90,m_ |
| **No ISC x ISC_SII_** | 7.4 ^-10^* | 3.18^-5^* |
| **ISC_SII_ x ISC_Noise_** | 0.107 | 0.45 |
| **No ISC x ISC_Noise_** | 5.47^-6^* | 9.18^-7^* |


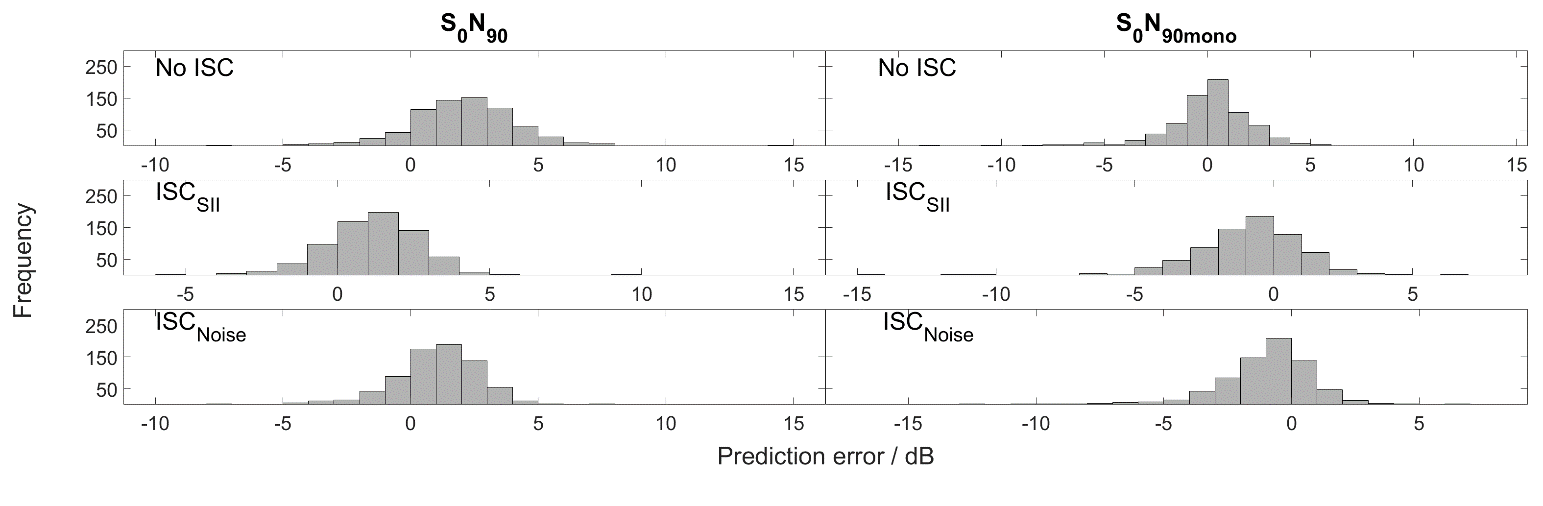


Figure S3: Histograms of prediction errors for the different SRT calculations and spatial conditions. The top panels display results without any individual component, the middle panels utilize an ISC based on individual SII references (ISC_SII_), and the bottom panels use an ISC based on an individually increased external noise (ISC_Noise_). The left column shows the results for S_0_N_90_ and the right column shows the results for S_0,m_N_90,m_. The S_0_N_0_ conditions are not shown, because when using the ISC, this condition is fitted and not predicted.

TABLE S2: p values of KS test of the different individualization methods and spatial conditions (S_0_N_0_, left; S_0_N_90_, middle; S_0,m_N_90,m_, right). The table shows the values without individualization in the top row, the values of the ISC_SII_ method in the middle row and the values of the ISC_Noise_ method in the bottom row. The p values for the S_0_N_0_ condition of the ISC_SII_ and the ISC_Noise_ individualization were not calculated because there is no prediction.

| **Manner of individualization** | **Spatial condition** | | |
| --- | --- | --- | --- |
|  | **S_0_N_0_** | **S_0_N_90_** | **S_0,m_N_90,m_** |
| **No ISC** | 0.9461 | 0.9573 | 0.0842 |
| **ISC_SII_** | No prediction | 0.6151 | 0.1678 |
| **ISC_Noise_** | No prediction | 0.7379 | 0.0743 |


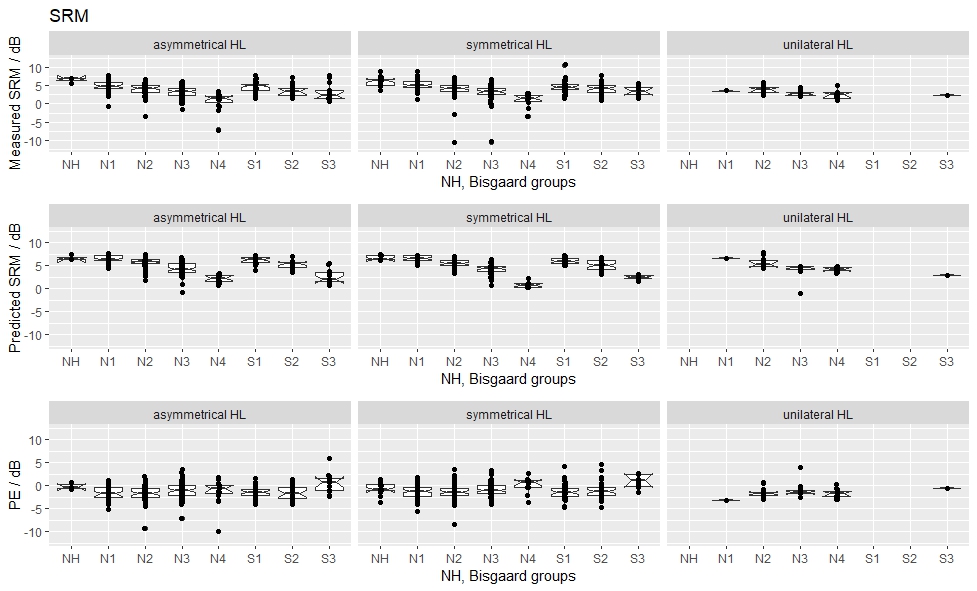


Figure S4: Comparison of measured (upper panels) and predicted SRM (middle panels) along with the prediction error (PE) between the measured and predicted data (lower panels). All results are categorized by NH and Bisgaard groups, as well as by HL symmetry: symmetrical (HL between left and right ear < 10 dB HL), asymmetrical (10 dB HL > HL between left and right ear < 40 dB HL), and unilateral HL (HL between left and right ear > 40 dB HL).
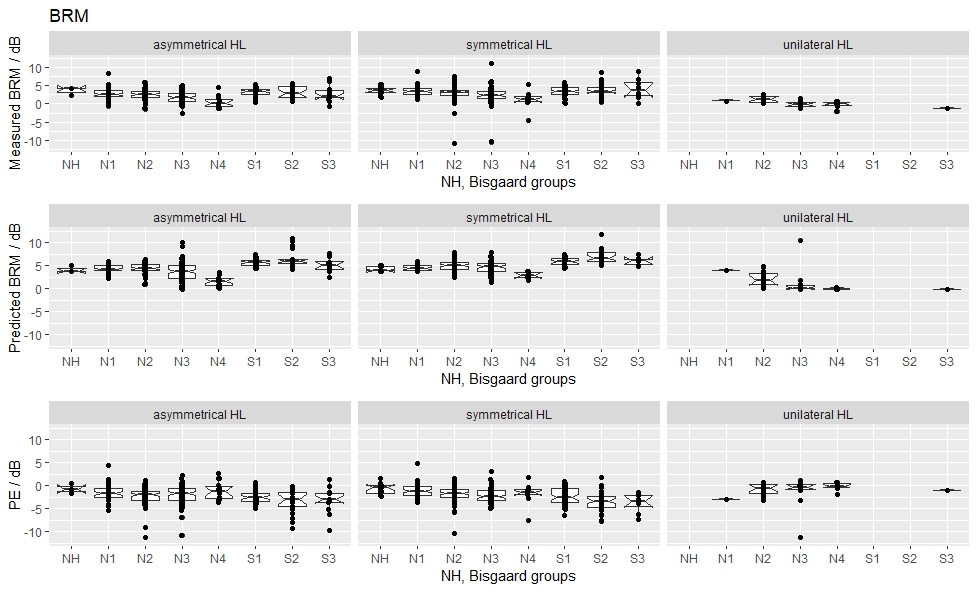


*Figure S5: Same as Figure S4 but for BRM. Here we see differences of the PE for the group S1, S2, and S3.*


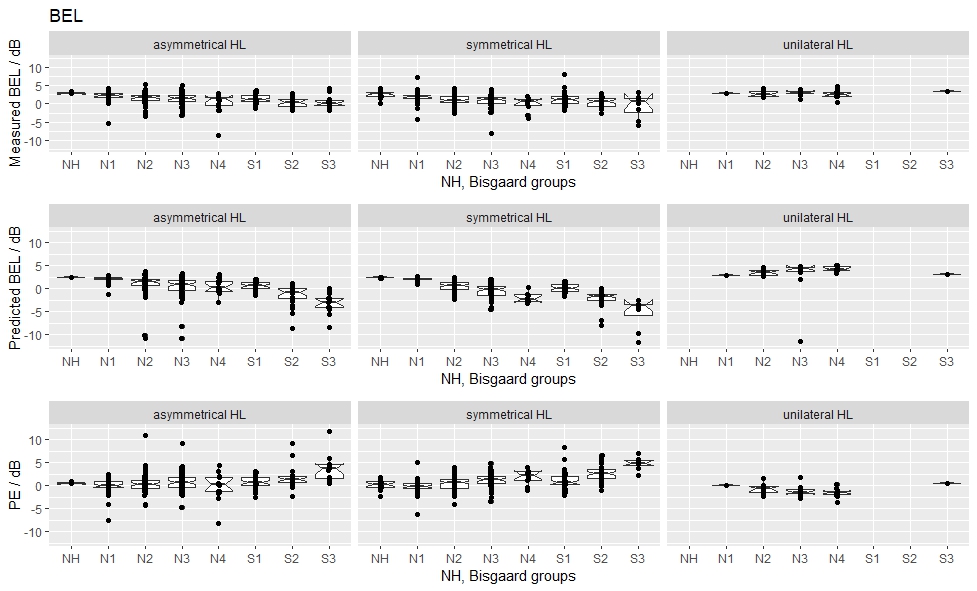


*Figure S6: Same as Figure S4 but for BEL. Here we see differences of the PE for the group S1, S2, and S3 into the other direction than for BRM.*


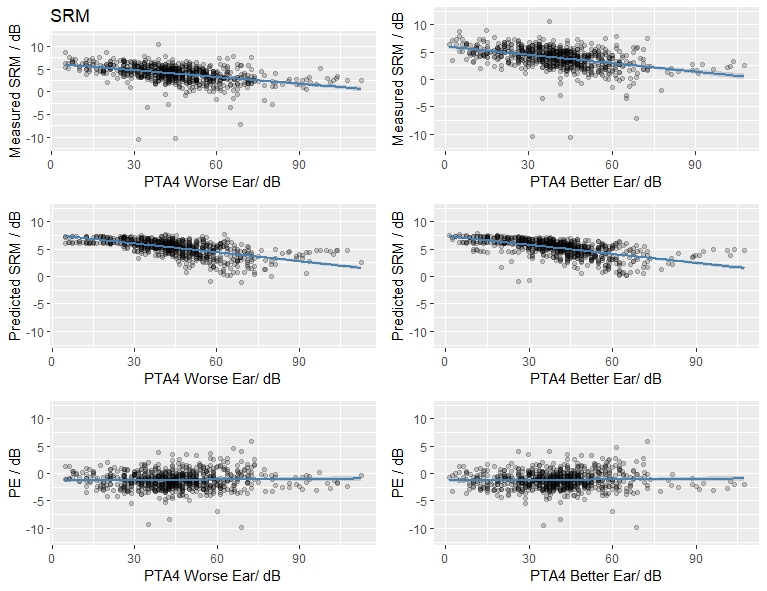


*Figure S7: Scatterplot of measured (upper panels) and predicted SRM (middle panels), along with the resulting prediction error (PE) (lower panels). The data are calculated once using the listener’s worse ear (left panels) and once using the listener’s better ear (right panels), including regression line (blue). SRM decreases with increasing PTA, which is also predicted by the model. The PE is nearly constant and independent of PTA*


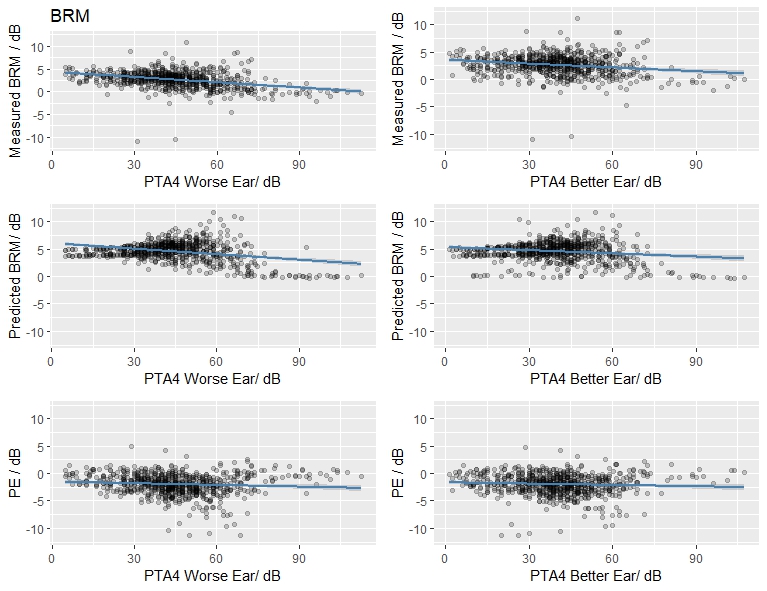


*Figure S8: Same as Figure S7, but for BRM.*


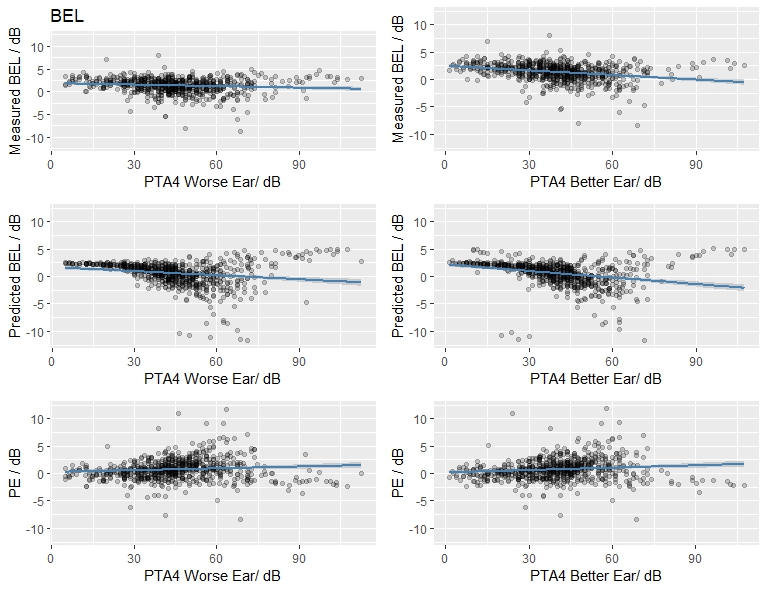


*Figure S9: Same as Figure 7 but for BEL. Here we see a small (r=0.1) but significant correlation of the PE. The data show a quite smooth decrease of BEL with increasing PTA, whereas the model predicts more an on or off effect. However, the correlation of PE with PTA is small and only significant due to the high statistical power of the data base.*

*TABLE S3: Statistical analysis of analysis of SRM, BRM, and BEL with respect to better and worse ear.*

| **Measure** | **Worse Ear** | **Better Ear** |
| --- | --- | --- |
| Measured SRM | r= -0.45; p-value < 2.2e-16 | r = -0.44; p-value < 2.2e-16 |
| Predicted SRM | r = -0.6; p-value < 2.2e-16 | r = -0.59; p-value < 2.2e-16 |
| PE SRM | r = 0.042; p-value = 0.25 | r = 0.035; p-value = 0.34 |
| Measured BRM | r= -0.35; p-value =<2.2e-16 | r = -0.205; p-value = 1.87e-08 |
| Predicted BRM | r = -0.25; p-value = 2.19e-12 | r = -0.13; p-value = 0.00049 |
| PE BRM | r = 0.078; p-value = 0.034 | r = -0.06; p-value = 0.095 |
| Measured BEL | r = -0.28; p-value = 1.18e-14 | r = -0.12; p-value = 0.001 |
| Predicted BEL | r = -0.195; p-value =8.8e-08 | r = 0.29; p-value = 2.27e-16 |
| PE BEL | r = 0.11; p-value = 0.002 | r = 0.089; p-value = 0.015 |

TABLE S4: One-sided pairwise comparison of the measured SRM for the different Bisgaard groups tests whether the mean of one group is significantly greater than the mean of the other group. P values were adjusted with a Bonferroni correction (Bonferroni correction = 0.0178) to maintain the significance level of 0.05.

|  | NH | N1 | N2 | N3 | N4 | S1 | S2 |
| --- | --- | --- | --- | --- | --- | --- | --- |
| N1 | 1.0 | - | - | - | - | - | - |
| N2 | 1.0 | 1.0 | - | - | - | - | - |
| N3 | 1.0 | 1.0 | 1.0 | - | - | - | - |
| N4 | 1.0 | 1.0 | 1.0 | 1.0 | - | - | - |
| S1 | 1.0 | 1.0 | 0.02554 | 2.8e-11 | < 2e-16 | - | - |
| S2 | 1.0 | 1.0 | 1.0 | 0.09172 | 6.4e-11 | 1.0 | - |
| S3 | 1.0 | 1.0 | 1.0 | 1.0 | 0.00054 | 1.0 | 1.0 |

TABLE S5: Same as TABLE S5 but for measured BRM.

|  | NH | N1 | N2 | N3 | N4 | S1 | S2 |
| --- | --- | --- | --- | --- | --- | --- | --- |
| N1 | 1.0 | - | - | - | - | - | - |
| N2 | 1.0 | 1.0 | - | - | - | - | - |
| N3 | 1.0 | 1.0 | 1.0 | - | - | - | - |
| N4 | 1.0 | 1.0 | 1.0 | 1.0 | - | - | - |
| S1 | 1.0 | 1.0 | 0.0072 | 2.3e-10 | < 2e-16 | - | - |
| S2 | 1.0 | 1.0 | 0.0172 | 2.7e-0.8 | 3.6e-16 | 1.0 | - |
| S3 | 1.0 | 1.0 | 1.0 | 0.0471 | 1.3e-07 | 1.0 | 1.0 |

TABLE S6: Same as TABLE S5 but for measured BEL.

|  | NH | N1 | N2 | N3 | N4 | S1 | S2 |
| --- | --- | --- | --- | --- | --- | --- | --- |
| N1 | 1.0 | - | - | - | - | - | - |
| N2 | 1.0 | 1.0 | - | - | - | - | - |
| N3 | 1.0 | 1.0 | 1.0 | - | - | - | - |
| N4 | 1.0 | 1.0 | 1.0 | 1.0 | - | - | - |
| S1 | 1.0 | 1.0 | 1.0 | 1.0 | 1.0 | - | - |
| S2 | 1.0 | 1.0 | 1.0 | 1.0 | 1.0 | 1.0 | - |
| S3 | 1.0 | 1.0 | 1.0 | 1.0 | 1.0 | 1.0 | 1.0 |

TABLE S7: Same as TABLE S5 but for predicted SRM.

|  | NH | N1 | N2 | N3 | N4 | S1 | S2 |
| --- | --- | --- | --- | --- | --- | --- | --- |
| N1 | 1.0 | - | - | - | - | - | - |
| N2 | 1.0 | 1.0 | - | - | - | - | - |
| N3 | 1.0 | 1.0 | 1.0 | - | - | - | - |
| N4 | 1.0 | 1.0 | 1.0 | 1.0 | - | - | - |
| S1 | 1.0 | 1.0 | 8.4e-0.5 | < 2e-16 | < 2e-16 | - | - |
| S2 | 1.0 | 1.0 | 1.0 | 2.9e-0.7 | < 2e-16 | 1.0 | - |
| S3 | 1.0 | 1.0 | 1.0 | 1.0 | 1.0 | 1.0 | 1.0 |

TABLE S8: Same as TABLE S5 but for predicted BRM.

|  | NH | N1 | N2 | N3 | N4 | S1 | S2 |
| --- | --- | --- | --- | --- | --- | --- | --- |
| N1 | 1.0 | - | - | - | - | - | - |
| N2 | 1.0 | 1.0 | - | - | - | - | - |
| N3 | 1.0 | 1.0 | 1.0 | - | - | - | - |
| N4 | 1.0 | 1.0 | 1.0 | 1.0 | - | - | - |
| S1 | 0.0025 | 5.4e-0.8 | 1.4e-0.7 | 5.9e-16 | < 2e-16 | - | - |
| S2 | 1.1e-0.9 | < 2e-16 | < 2e-16 | < 2e-16 | < 2e-16 | 3e-0.5 | - |
| S3 | 0.0007 | 1.1e-0.5 | 0e-0.5 | 5.7e-0.9 | < 2e-16 | 1.0 | 1.0 |

TABLE S9: Same as TABLE S5 but for predicted BEL.

|  | NH | N1 | N2 | N3 | N4 | S1 | S2 |
| --- | --- | --- | --- | --- | --- | --- | --- |
| N1 | 1.0 | - | - | - | - | - | - |
| N2 | 1.0 | 1.0 | - | - | - | - | - |
| N3 | 1.0 | 1.0 | 1.0 | - | - | - | - |
| N4 | 1.0 | 1.0 | 1.0 | 0.046 | - | - | - |
| S1 | 1.0 | 1.0 | 1.0 | 1.0 | 1.0 | - | - |
| S2 | 1.0 | 1.0 | 1.0 | 1.0 | 1.0 | 1.0 | - |
| S3 | 1.0 | 1.0 | 1.0 | 1.0 | 1.0 | 1.0 | 1.0 |

TABLE S10: One-sided pairwise comparison of the measured SRM for the different Bisgaard groups tests whether the mean of one group is significantly smaller than the mean of the other group. P values were adjusted with a Bonferroni correction (Bonferroni correction = 0.0178) to maintain the significance level of 0.05.

|  | NH | N1 | N2 | N3 | N4 | S1 | S2 |
| --- | --- | --- | --- | --- | --- | --- | --- |
| N1 | 0.505 | - | - | - | - | - | - |
| N2 | 9.8e-08 | 4.6e-07 | - | - | - | - | - |
| N3 | 2.8e-14 | < 2e-16 | 9.2e-06 | - | - | - | - |
| N4 | < 2e-16 | < 2e-16 | < 2e-16 | 8.1e08 | - | - | - |
| S1 | 0.0013 | 0.9065 | 1.0 | 1.0 | 1.0 | - | - |
| S2 | 2.4e-07 | 3.8e-05 | 1.0 | 1.0 | 1.0 | 0.0383 | - |
| S3 | 7.7e-08 | 3.1e-05 | 0.5548 | 1.0 | 1.0 | 0.0047 | 1.0 |

TABLE S11: Same as TABLE S11 but for measured BRM.

|  | NH | N1 | N2 | N3 | N4 | S1 | S2 |
| --- | --- | --- | --- | --- | --- | --- | --- |
| N1 | 1.0 | - | - | - | - | - | - |
| N2 | 0.13538 | 0.13944 | - | - | - | - | - |
| N3 | 0.00011 | 1.2e-08 | 0.00043 | - | - | - | - |
| N4 | 3.6e-11 | 2.4e-16 | 3.0e-12 | 1.1e-05 | - | - | - |
| S1 | 1.0 | 1.0 | 1.0 | 1.0 | 1.0 | - | - |
| S2 | 1.0 | 1.0 | 1.0 | 1.0 | 1.0 | 1.0 | - |
| S3 | 1.0 | 1.0 | 1.0 | 1.0 | 1.0 | 1.0 | 1.0 |

TABLE S12: Same as TABLE S11 but for measured BEL.

|  | NH | N1 | N2 | N3 | N4 | S1 | S2 |
| --- | --- | --- | --- | --- | --- | --- | --- |
| N1 | 1.0 | - | - | - | - | - | - |
| N2 | 0.01070 | 0.02787 | - | - | - | - | - |
| N3 | 0.00307 | 0.00305 | 1.0 | - | - | - | - |
| N4 | 0.00319 | 0.02198 | 1.0 | 1.0 | - | - | - |
| S1 | 0.00832 | 0.03613 | 1.0 | 1.0 | 1.0 | - | - |
| S2 | 5.3e-07 | 1.2e-08 | 0.00034 | 0.00387 | 0.79782 | 0.0778 | - |
| S3 | 1.3e-05 | 4.1e-05 | 0.01568 | 0.05449 | 0.85946 | 0.05861 | 1.0 |

TABLE S13: Same as TABLE S11 but for predicted SRM.

|  | NH | N1 | N2 | N3 | N4 | S1 | S2 |
| --- | --- | --- | --- | --- | --- | --- | --- |
| N1 | 1.0 | - | - | - | - | - | - |
| N2 | 1.7e-06 | 1.4e-15 | - | - | - | - | - |
| N3 | < 2e-16 | < 2e-16 | < 2e-16 | - | - | - | - |
| N4 | < 2e-16 | < 2e-16 | < 2e-16 | < 2e-16 | - | - | - |
| S1 | 0.104 | 0.039 | 1.0 | 1.0 | 1.0 | - | - |
| S2 | 2.7e-10 | < 2e-16 | 0.01 | 1.0 | 1.0 | 2.6e-09 | - |
| S3 | < 2e-16 | < 2e-16 | < 2e-16 | 2.5e-12 | 1.0 | < 2e-16 | < 2e-16 |

TABLE S14: Same as TABLE S11 but for predicted BRM.

|  | NH | N1 | N2 | N3 | N4 | S1 | S2 |
| --- | --- | --- | --- | --- | --- | --- | --- |
| N1 | 1.0 | - | - | - | - | - | - |
| N2 | 1.0 | 1.0 | - | - | - | - | - |
| N3 | 1.0 | 0.2417 | 0.0047 | - | - | - | - |
| N4 | 1.7e-10 | < 2e-16 | < 2e-16 | < 2e-16 | - | - | - |
| S1 | 1.0 | 1.0 | 1.0 | 1.0 | 1.0 | - | - |
| S2 | 1.0 | 1.0 | 1.0 | 1.0 | 1.0 | 1.0 | - |
| S3 | 1.0 | 1.0 | 1.0 | 1.0 | 1.0 | 1.0 | 1.0 |

TABLE S15: Same as TABLE S11 but for predicted BEL.

|  | NH | N1 | N2 | N3 | N4 | S1 | S2 |
| --- | --- | --- | --- | --- | --- | --- | --- |
| N1 | 1.0 | - | - | - | - | - | - |
| N2 | 0.00462 | 1.4e-06 | - | - | - | - | - |
| N3 | 1.3e-06 | < 2e-16 | 0.00112 | - | - | - | - |
| N4 | 0.13849 | 0.10127 | 1.0 | 1.0 | - | - | - |
| S1 | 2.2e-05 | 9.3e-11 | 0.10554 | 1.0 | 0.26550 | - | - |
| S2 | < 2e-16 | < 2e-16 | < 2e-16 | 1.4e-13 | 1.0e-14 | 9.0e-13 | - |
| S3 | < 2e-16 | < 2e-16 | < 2e-16 | < 2e-16 | < 2e-16 | < 2e-16 | 0.00066 |
